# Relationship between the washout rate of I-123 MIBG scans and autonomic function in Parkinson’s disease

**DOI:** 10.1371/journal.pone.0229860

**Published:** 2020-03-05

**Authors:** Young Jin Jeong, Ji-Eun Jeong, Sang-Myung Cheon, Byeol-A Yoon, Jae Woo Kim, Do-Young Kang

**Affiliations:** 1 Departments of Nuclear Medicine, Dong-A University Hospital, Dong-A University College of Medicine, Busan, Republic of Korea; 2 Institute of Convergence Bio-Health, Dong-A University, Busan, Republic of Korea; 3 Departments of Neurology, Dong-A University Hospital, Dong-A University College of Medicine, Busan, Republic of Korea; Oslo Universitetssykehus, NORWAY

## Abstract

**Purpose:**

We have evaluated the clinical significance of the washout rate (WR) on I-123 MIBG scans through the analysis of the relationship between the I-123 MIBG scans and autonomic status in patients with Parkinson’s disease (PD).

**Materials and methods:**

Sixty patients with clinical PD who had decreased HMR were enrolled. An autonomic symptom was evaluated using a head-up tilt test and the Composite Autonomic Severity Score (CASS). An I-123 MIBG scan and F-18 FP-CIT positron emission tomography (PET) were performed. All of the patients were classified into three groups according to the WR. The differences in patient characteristics and the imaging parameters among the three groups were evaluated, and a correlation analysis was also performed.

**Results:**

The frequency of orthostatic hypotension was significantly different among the three groups. The difference in systolic pressure (dSysPr) and the difference in diastolic pressure (dDiaPr) of group 3 was significantly larger than those of groups 1 and 2. From the correlation analysis, it can be seen that age, Hoehn and Yahr (H&Y) stage, dSysPr, and dDiaPr had a weak positive correlation with the WR. The total CASS score was significantly higher in group 3 compared with groups 1 and 2. The WR had a moderate positive correlation with the cardiosympathetic score and the total CASS score.

**Conclusion:**

The WR is related to autonomic dysfunction. An I-123 MIBG cardiac scan is considered to be a good method to evaluate not only the differential diagnosis of Parkinson's disease but also the degree of autonomic dysfunction.

## Introduction

Lewy body diseases including Parkinson’s disease (PD) are characterized by a motor symptom such as resting tremors, rigidity and bradykinesia due to dopaminergic neural degeneration; cardiac autonomic failure due to sympathetic neuronal degeneration is also a common feature of the disease [1, 2]. In PD, a lot of attention has been placed on assessing autonomic failure because clinical signs of related autonomic failure may observe in the early phase of the disease, or even before the onset of the motor symptoms [2–5].

Metaiodobenzylguanidine (MIBG) is a physiologic analog of guanethidine and its uptake and storage mechanism is similar to that of noradrenaline, which is actively transported into noradrenaline granules in the sympathetic nerve terminals by noradrenaline transport [6]. In general, cardiac uptake in I-123 MIBG cardiac scans is known to decrease in patients with PD, and in one study, decreased myocardial uptake was observed in approximately 90% of patients with PD [7, 8]. Therefore, I-123 MIBG cardiac scans have been widely used to assess non-invasively the presynaptic cardiac sympathetic nerve endings. Many studies have focused on the differential diagnosis of PD from atypical parkinsonism, such as multiple system atrophy or corticobasal degeneration [9–17]. Those studies consistently showed that I-123 MIBG cardiac scans were very useful in distinguishing early PD, and that the heart-to-mediastinal ratio (HMR) was a good diagnostic indicator in discriminating those diseases. The HMR, a quantification parameter of cardiac I-MIBG uptake, is known to reflect receptor density and to show the integrity of presynaptic nerve terminals [18]. A reduction of the HMR has also been reported to be related to dementia development, disease progression, motor severity, or clinical phenotypes in patients with PD [8, 19–22].

From I-123 MIBG cardiac scans, another quantification measurement that can be obtained is the washout rate (WR), which is a ratio of cardiac uptake between early and delayed scans. The WR is thought to reflect the turnover of catecholamines and sympathetic tone [23, 24]. It means that an increased WR indicates worsening cardiac function, and this has been revealed in studies of patients with heart failure. In those studies, patients who had heart failure with an increased WR had an increased cardiac death rate or hospital admission [25]. In PD, while the usefulness of the HMR has been well studied in I-123 MIBG scans, the clinical significance or the role of the WR has not been clearly elucidated. Some studies have revealed that the WR was not related to activities of daily living, motor symptoms, or the dose of medication [21, 26]. The HMR is a good parameter to discriminate Parkinsonism and evaluate various clinical situations, but many clinical studies have shown that a reduced HMR was not always related to autonomic symptoms such as orthostatic hypotension (OH) [8, 27–29]. In PD, autonomic dysfunction is one of the most frequent non-motor symptoms, and evaluation of the dysfunction is important because it may precede motor symptoms. Because the WR is considered to be related to sympathetic tone, we considered the possibility of the relationship between the WR and autonomic dysfunction.

In the present study, we have evaluated the clinical significance of the WR from I-123 MIBG scans through the analysis of the relationship between the I-123 MIBG scans and autonomic function in patients with PD.

## Materials and methods

### Patients

Patients with PD who performed autonomic function test, I-123 MIBG scan and F-18 FP-CIT PET between January 2017 and May 2018 were reviewed retrospectively. Among the patients, we enrolled subjects who had decreased delayed heart-to-mediastinal ratios (dHMR) lower than the cutoff value from the I-123 MIBG scans, to evaluate the clinical significance of the WR. The diagnosis of the disease was made by movement disorder specialists based on the UK Parkinson’s Disease Society Brain Bank Clinical Diagnosis Criteria. The neurologists checked for motor disability, such as Parkinsonism (*e*.*g*. tremors, rigidity, bradykinesia and postural instability) and the Unified Parkinson’s disease rating scale (UPDRS) was used to assess the severity of the disease. An autonomic symptom was also evaluated using a head-up tilt (HUT) test in all patients. In patients taking dopaminergic medication for the treatment of Parkinsonism, doses of the drugs were investigated and expressed as levodopa equivalent doses (LEDs) [18]. I-123 MIBG scans and F-18 FP-CIT PET were performed on all patients, and the time interval between the two imaging studies was within two weeks. Because several diseases can affect the I-123 MIBG uptake, patients who had diabetes mellitus, clinical history of the cardiac disease (ex. arrhythmia or ischemic heart disease), and a sign of cardiac disease on electrocardiography were excluded. The patients with a history of head trauma, strokes, dementia, or psychological disorders were also excluded. The Institutional Review Board of Dong-A University Hospital reviewed and approved the study protocol (DAUHIRB-19-031). The written informed consent was obtained from all individual participants included in the study.

### Autonomic function test

In all patients, the HUT test using a tilt table was performed to assess autonomic function. During the HUT test, systolic and diastolic blood pressure, and heart rate were measured using a sphygmomanometer at 1-min intervals. OH was defined as a decrease in the systolic blood pressure of at least 20 mmHg or the diastolic blood pressure of at least 10 mmHg within 3 min of tilting.

The CASS is a validated scoring system of autonomic deficits derived from postganglionic sudomotor, cardiosympathetic, and cardiovagal function. Results are compared to a normal database. A 10-point scoring system of the CASS is generated that corrects for the effects of age and gender. The three subscales are adrenergic (range = 0–4), sudomotor (range = 0–3), and cardiovagal (range = 0–3). Generally, a total CASS score ≤ 3 indicates no or mild autonomic failure, scores from 4–6 indicate moderate autonomic failure, and scores from 7–10 indicate severe autonomic failure.

### F-18 FP-CIT PET/CT and image analysis

All of the F-18 FP-CIT PET/CT examinations were performed using a Biograph mCT flow (Siemens Healthcare, Knoxville, TN, USA) scanner. Patients were intravenously injected with 185 MBq F-18 FP-CIT, and PET/CT acquisition was started 120 min after the radiotracer injection. A helical CT scan was carried out with a rotation time of 0.5 s at 120 kVp and 100 mAs without an intravenous contrast agent. A PET scan followed immediately afterward and the scan was acquired for 10 min in the three-dimensional mode. All of the images were acquired from the skull vertex to the base of the skull. The patients were allowed to continue their anti-Parkinson medication.

Two experienced nuclear medicine physicians reviewed all of the PET/CT images using a dedicated workstation with custom software (Syngo.Via, VB20A_HF05). The striatal volumetric analysis was done following the protocol of a previous study [30]. To analyze the striatal functional volume (SFV), a semi-automatic delineated spherical volume-of-interest (VOI) was drawn over each of the two strata. The striatal target volume was segmented using an isocontour method. We drew the VOI over the occipital lobe, and the value of the SFV multiplied by the occipital mean standardized uptake value (SUVmean) was considered as the non-specific uptake of the striatum. Specific uptake ratios (SURs) were calculated for the target striatal VOI, and these values were defined as follows: (SUVmean of striatal VOI–SUVmean of occipital VOI)/ SUVmean of occipital VOI.

### Cardiac I-123 MIBG scans and image analysis

Early and delayed cardiac MIBG scans were performed at 30 min and 3 h after the intravenous injection of 111 MBq of I-123 MIBG, respectively. The planar image was obtained with a dual-head gamma camera equipped with a low-energy high-resolution parallel-hole collimator (eCAM, Siemens Healthcare, Knoxville, TN, USA). Medications that could affect MIBG uptake were withheld for 24 hours prior to the administration of I-123 MIBG. Relative cardiac uptake was determined by setting the region-of-interest (ROI) on the anterior view. The entire left ventricular ROI was drawn manually and a rectangular ROI was also set on the upper mediastinum area. The HMR of the early (eHMR) and delayed (dHMR) images were calculated by dividing the average count density per pixel of the left ventricular ROI by that of the mediastinal ROI, respectively. Normal HMR was defined as greater than 1.8 in the delayed image, a number based on a previous study [31]. We also calculated the WR, which is an index of the rate at which MIBG is washed out between the early image and the delayed image, according to the following formula;

WR (%) = [(early cardiac uptake–early mediastinal uptake)–(delayed cardiac uptake–delayed mediastinal uptake) × 1.21] × 100 / (early cardiac uptake–early mediastinal uptake)

The factor 1.21 is multiplied by the delayed value to correct for I-123 decay at 3 hours [25].

### Statistical analysis

We classified the subjects into three groups according to the WR in I-123 MIBG scans (Group 1: WR ≤ 20%, Group 2: 20% < WR ≤ 40% and Group 3: WR > 40%). The differences in the patient characteristics and imaging parameters among the three groups were evaluated using one-way ANOVA or the Kruskal-Wallis test with post hoc analysis for continuous variables and the Chi-squared or Fisher-Freeman-Halton exact test for categorical variables. The relationship between the WR and various parameters was evaluated using Spearman’s rho. The cutoff value of the WR for assessing OH was analyzed according to the receiver-operating characteristic (ROC) curve. In particular, sensitivity, positive predictive value (PPV), negative predictive value (NPV), area under the curve (AUC), and 95% confidence interval (CI) were calculated after optimization of the cutoff value by ROC analysis. In the 20 patients who had a CASS test, we also did the same analysis. The statistical analyses were performed using NCSS 12 Statistical Software (NCSS, LLC. Kaysville, Utah, USA) or MedCalc software version 16.4 (MedCalc Software, Mariakerke, Belgium). Statistical significance was defined as a p-value < 0.05.

## Results

### Patient characteristics (Table 1)

Sixty patients with clinically PD (M:F = 35:25, 68.5 ± 7.0 years) were enrolled retrospectively in this study. Various Hoehn & Yahr (H&Y) stages and UPDRS scores were seen in the enrolled subjects, and H&Y stage 2 was the most frequent (68.3%). From the I-123 MIBG scan, the dHMR of all the subjects was lower than 1.8, and the mean dHMR was 1.31 ± 0.15 (range: 1.17–1.72). A wide range of the WR was seen, and the mean value was 29.0% ± 18.2% (range: -1.9%– 69.8%). In this study, 32 patients (53.3%) were diagnosed as OH from the HUT test. The difference of the systolic and diastolic blood pressure between the supine and head-up tilt position was 22.5 ± 17.1 and 7.0 ± 10.7, respectively. From the F-18 FP-CIT PET, the SFV and SUR were 10.3 ± 3.0 and 4.0 ± 1.2, respectively. Comparing with a previous study at our institution [31], both PET parameters were all lower than those of healthy subjects.

**Table 1:** Patients characteristics and imaging parameters.

Characteristics	Total (n = 60)
Demographics	
Age, years	68.5 ± 7.0 (range: 54–87)
Male, n (%)	35 (58.3)
H&Y stage	2.2 ± 0.4
Stage 1	0
Stage 1.5	3
Stage 2	41
Stage 2.5	7
Stage 3	9
UPDRS	43.8 ± 19.5 (range: 14–89.5)
Disease duration, years	3.1 ± 2.3 (range: 0.5–12.7)
Orthostatic hypotension, n (%)	32 (53.3)
dSysPr (mmHg)	22.5 ± 17.1 (range: -6–80)
dDiapr (mmHg)	7.0 ± 10.7 (range: -11–40)
dHR	4.2 ± 8.0 (range: -12–28)
MMSE	26.0 ± 3.4 (range: 16–30)
LED, mg	324.1 ± 312.4 (range: 50–1680)
F-18 FP-CIT PET	
SFV	10.3 ± 3.0 (range: 5.1–17.5)
SUR	4.0 ± 1.2 (range: 1.8–6.3)
I-123 MIBG scan	
eHMR	1.39 ± 0.15 (range: 1.08–1.77)
dHMR	1.31 ± 0.15 (range: 1.17–1.72)
WR, %	29.0 ±18.2 (range: -1.9–69.8)

Values represent the mean ± standard deviation or number of subjects (percentage).

### Group comparison among the three groups and correlation analysis

**Fig 1:**
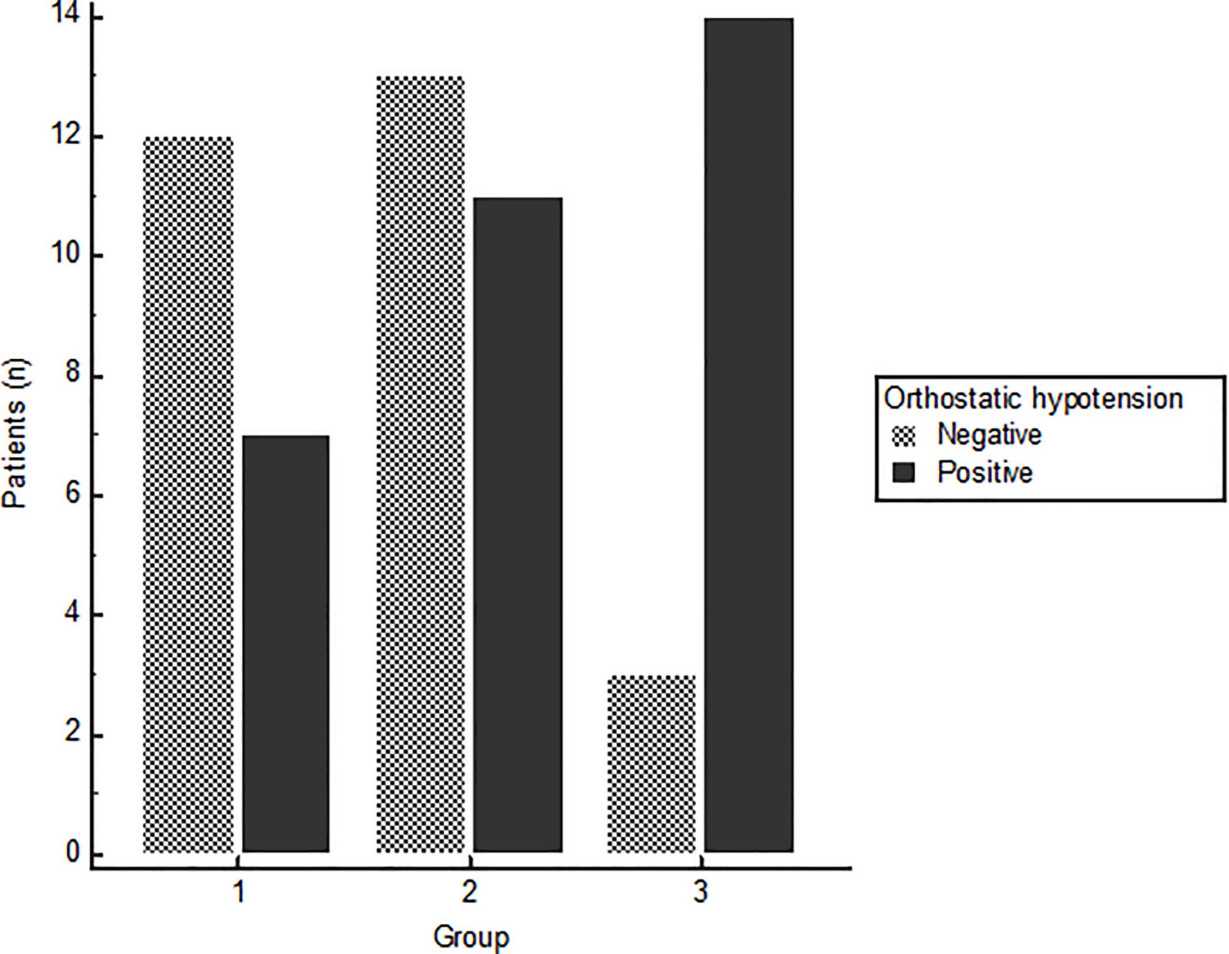
Frequency of orthostatic hypotension. The frequency of orthostatic hypotension is highest in group 3 and tends to increase from group 1 to group 3.

**Fig 2:**
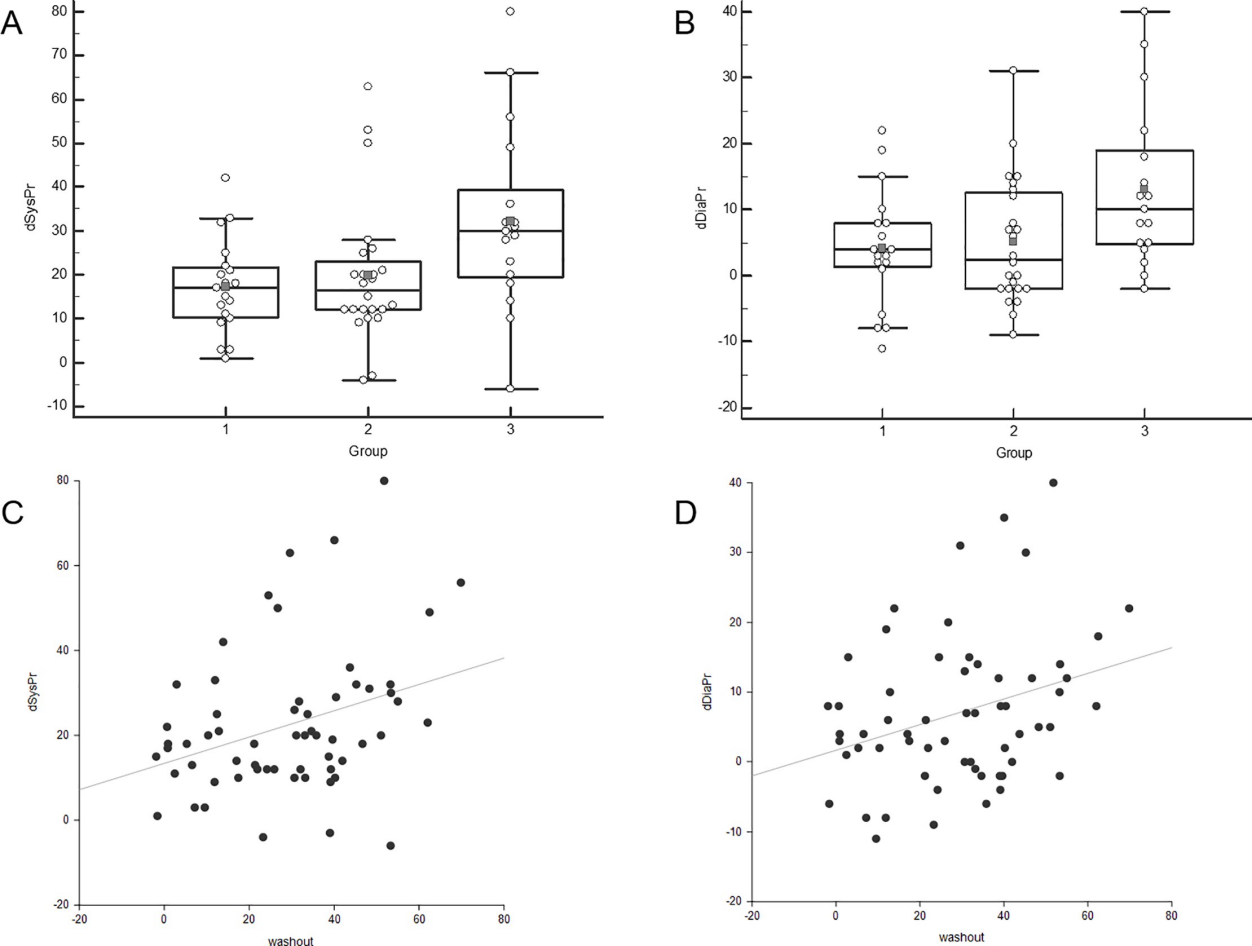
Comparison of the difference of systolic and diastolic pressure. A significant difference is shown in the difference of systolic pressure (A) and the difference of diastolic pressure (B) amongst the three groups. These two parameters also weakly correlated with the WR in all subjects (C, D).

From the 60 patients, 19 (31.7%) were in group 1, 24 (40.0%) were in group 2, and 17 (28.3%) were in group 3 (Table 2). There was a significant difference in age and H&Y stage, and age was significantly higher in groups 2 and 3 compared to group 1 (p = 0.025), and the H&Y stage was significantly higher in group 3 compared to group 1 (p = 0.012). The frequency of OH was significantly different among the three groups (p = 0.011, Fig 1). Although the difference of heart rate (dHR) was not different between the groups, the difference of systolic pressure (dSysPr) (p = 0.016) and the difference of diastolic pressure (dDiaPr) (p = 0.018) of group 3 was significantly larger than those of group 1 and 2 (Fig 2A and 2B). There was no significant difference in the disease duration, UPDRS, MMSE, and LED. Next, the analysis of covariance (ANCOVA) was performed with age and H&Y stage as a covariate, and the dSysPr and dDiaPr were still significantly higher in group 3 than the other groups (p = 0.034 and p = 0.028).

**Table 2:** Difference between clinical and imaging parameters among the three groups.

Characteristics	Group 1	Group 2	Group 3	P-value
	(n = 19)	(n = 24)	(n = 17)	
Demographics				
Age, years	64.9 ± 6.2	70.1 ± 6.9	70.1 ± 6.8	***0*.*025 (1<2*, *3)***^a^
Male, n (%)	15 (78.9)	10 (41.7)	10 (58.8)	***0*.*048***^b^
H&Y stage	2.0 ± 0.1	2.2 ± 0.4	2.4 ± 0.5	***0*.*012 (1<3)***^a^
Stage 1	0	0	0	
Stage 1.5	1	2	0	
Stage 2	18	13	10	
Stage 2.5	0	5	2	
Stage 3	0	4	5	
UPDRS	47.0 ± 22.4	42.8 ± 11.7	41.5 ± 25.0	0.825
Disease duration, years	2.7 ± 1.2	3.2 ± 2.3	3.5 ± 3.2	0.137
Orthostatic hypotension, n (%)	7 (36.8)	11 (45.8)	14 (82.4)	***0*.*011***^b^
dSysPr	17.2 ±10.7	19.7 ±15.9	32.2 ±20.9	***0*.*016 (1*, *2<3)***^a^
dDiapr	4.1 ± 8.7	5.0 ± 9.5	13.1 ± 12.2	***0*.*018 (1*, *2<3)***^a^
dHR	3.6 ± 8.6	6.3 ± 7.0	1.8 ± 8.4	0.200
MMSE	27.5 ± 2.1	25.3 ± 3.9	25.3 ±3.5	0.077
LED, mg	365.7 ± 313.0	303.2 ± 229.0	307.1 ± 413.8	0.450
F-18 FP-CIT PET				
SFV	11.0 ± 2.9	9.8 ± 3.0	10.3 ± 3.1	0.455
SUR	4.0 ± 1.1	4.1 ± 1.4	3.6 ± 1.0	0.436
I-123 MIBG scan				
eHMR	1.40 ± 0.14	1.36 ± 0.18	1.42 ± 0.12	0.517
dHMR	1.38 ± 0.18	1.28 ± 0.15	1.28 ± 0.09	0.074
WR, %	7.4 ± 6.2	30.9 ± 6.1	50.5 ± 8.6	***<0*.*001 (1<2<3)***^a^

Values represent the mean ± standard deviation or number of subjects (percentage).

^a^parentheses indicated a result of post hoc analysis using the Scheffe’s test

^b^Chi-squared test

From the I-123 MIBG scans, there was a significant difference in the WR (7.4% ± 6.2% in group 1, 30.9% ± 6.1% in group 2 and 50.5% ± 8.6% in group 3, p < 0.001), but not in the eHMR and dHMR (p = 0.517 and 0.074). From the F-18 FP-CIT PET scans, no significant difference was revealed in the SFV and SUR among the three groups (p = 0.455 and 0.436).

In the correlation analysis, age (r = 284, p = 0.028), H&Y stage (r = 0.386, p = 0.002), dSysPr (r = 0.320, p = 0.013) and dDiaPr (r = 0.275, p = 0.033) had a weak positive correlation with the WR in all subjects (Table 3, Fig 2C and 2D). The WR was not significantly correlated with the disease duration, UPDRS, disease duration, MMSE, LED, dHR, SFV, and SUR, respectively.

**Table 3:** Correlation between washout rate and patient characteristics.

Characteristics	Correlation coefficient (r)	P-value
Age, years	0.284	***0*.*028***
Orthostatic hypotension, n (%)		
dSysPr (mmHg)	0.320	***0*.*013***
dDiapr (mmHg)	0.275	***0*.*033***
dHR	-0.045	0.733
H&Y stage	0.386	***0*.*002***
UPDRS	-0.121	0.524
Disease duration	0.022	0.866
MMSE	-0.251	0.053
LED, mg	-0.164	0.210
F-18 FP-CIT PET		
SFV	-0.125	0.360
SUR	-0.136	0.318

In ROC curve analysis, the WR of 26% from the I-123 MIBG cardiac scans was found to be a reliable indicator to assess OH with an area under the curve of 0.725 (p = 0.001, 95% CI = 0.595–0.833). The sensitivity was 75.0%, specificity was 65.3%, the PPV was 69.4%, and the NPV was 70.8%.

### Group comparison and correlation analysis between the washout rate and CASS

**Fig 3:**
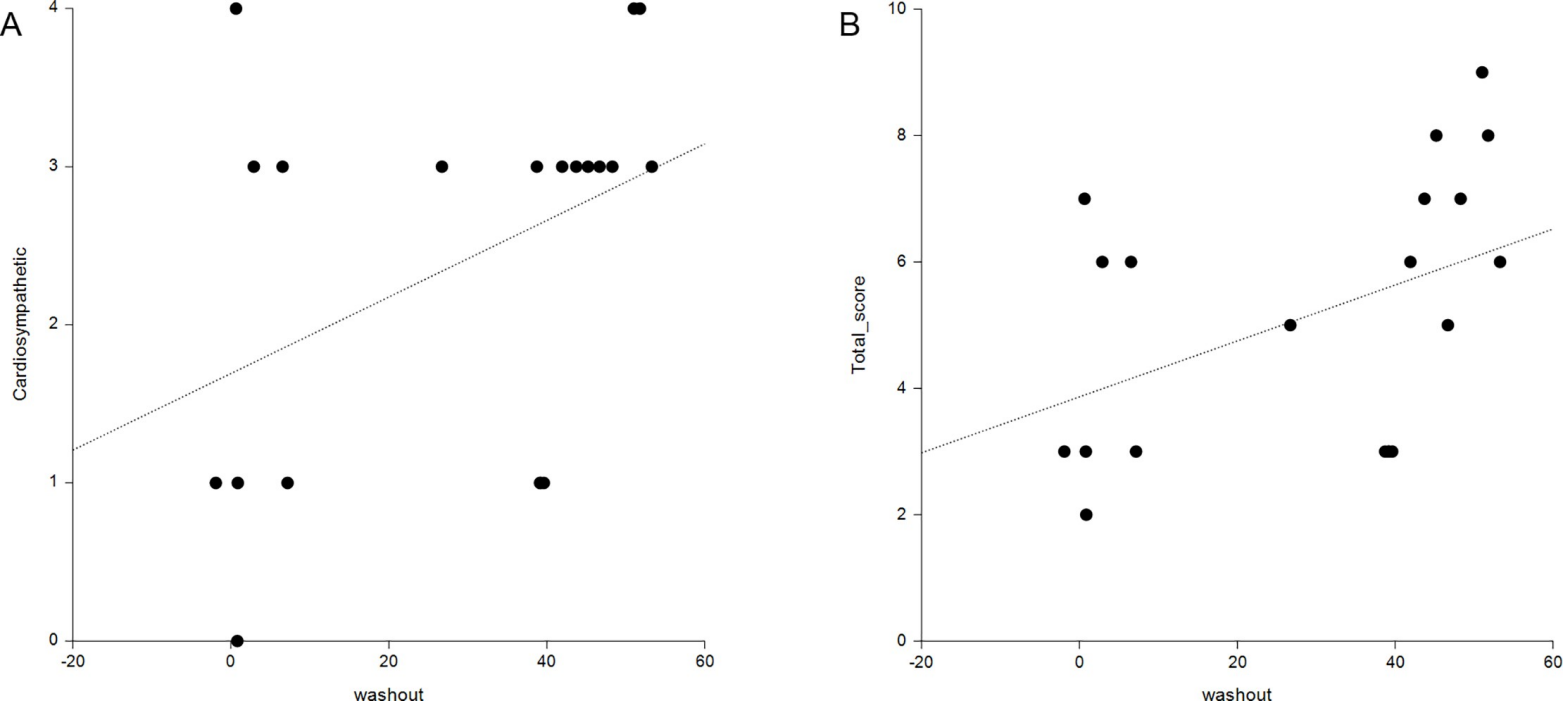
Correlation analysis between CASS score and washout rate. In the CASS test, the WR is moderately correlated with the cardiosympathetic (A) and total (B) scores in twenty patients with PD.

A total of 60 patients were recommended for a further evaluation using the Composite Autonomic Severity Score (CASS) for precise quantification of an autonomic function, and 20 patients agreed to the test. We also did a group comparison, and correlation analysis between the WR and the CASS score in the 20 patients who performed the CASS test (Tables 4 and 5). Of the 20 patients, 7 (35.0%) were in group 1, 5 (25.0%) were in group 2, and 8 (40.0%) were in group 3. The total CASS score was significantly higher in group 3 compared with groups 1 and 2 (p = 0.006). The CASS grade was also significantly different among the three groups (p = 0.027). The cardiosympathetic score was higher in group 3, compared with those of groups 1 and 2, but the result did not achieve statistical significance (p = 0.051). There was no significant difference in the sudomotor and cardiovagal scores. In the correlation test, the WR had a moderate positive correlation with the cardiosympathetic score (r = 0.456, p = 0.043) and the total CASS score (r = 0.555, p = 0.011) (Fig 3A and 3B).

**Table 4:** Difference of CASS-related variables among the three groups (n = 20).

Characteristics	Group 1	Group 2	Group 3	P-value
	(n = 7)	(n = 5)	(n = 8)	
Sudomotor	1.3 ± 0.5	1.0 ± 0.9	1.9 ± 0.6	0.094
Cardiovagal	1.1 ± 0.9	0.8 ± 0.8	1.9 ± 1.0	0.124
Cardiosympathetic	1.9 ± 1.5	1.8 ± 1.1	3.3 ± 0.5	0.051
Total score	4.3 ± 1.9	3.4 ± 0.9	7.0 ± 1.3	***0*.*006 (3>1*, *2***^***)***^^***a***^
CASS grade				***0*.*014***^***b***^
Mild	4	4	0	
Moderate	2	1	3	
Severe	1	0	5	

Values represent the mean ± standard deviation or number of subjects.

^a^parentheses indicated a result of post hoc analysis using the Mann-Whitney test with Bonferroni correction method

^b^Fisher-Freeman-Halton exact test

**Table 5:** Correlation between washout rate and CASS-related variables (n = 20).

Characteristics	Correlation coefficients	P-value
Sudomotor	0.273	0.231
Cardiovagal	0.383	0.095
Cardiosympathetic	0.456	***0*.*043***
Total score	0.555	***0*.*011***

To evaluate the relationship between the dHMR and autonomic function, a correlation analysis was performed. There was no significant correlation between the dHMR and autonomic function parameters, including dSysPr (r = -0.223, p = 0.087), dDiaPr (r = -0.236, p = 0.070), dHR (r = 0.113, p = 0.391), sudomotor (r = -0.280, p = 0.218), cardiovagal (r = -0.076, p = 0.749), cardiosympathetic (r = -0.251, p = 0.287), total CASS score(r = -0.272, p = 0.246), and CASS grade (r = -0.243, p = 0.303).

## Discussion

In the present study, the WR had a weak correlation with OH related factors, and a moderate correlation with the CASS, and thus we found that there was a relationship between autonomic function and the WR of the I-123 MIBG cardiac scans. Sympathetic hyperactivation is related to a suppression of the cardio-inhibitory reflexes and induces an increased turnover of catecholamine up to 50-fold compared to that in controls; therefore, sustained sympathicotonia contributes to worsening cardiac function [32]. Because an increased WR reflects a rapid turnover of norepinephrine by sympathetic drive or a decreased ability to preserve norepinephrine in cardiac sympathetic terminals, we proposed that the WR had a correlation with autonomic function parameters in this study. There have not been many studies on this topic, one study revealed that the dSysPr (r = 0.34, p = 0.02) and the dDiaPr (r = 0.29, p = 0.04) were weakly correlated with only the WR, but not correlated with the eHMR and the dHMR, which was similar to our result [26]. The HMR was not associated with autonomic parameters in this study. Most previous studies that analyzed the correlation between I-123 MIBG cardiac scans and autonomic function used the HMR as an imaging parameter, and reported inconsistent results [33–35]. Berganzo K, *et al*. reported that no significant correlation was found between the HMR and scores on the autonomic function scale [34]. It is thought that the sensitivity of the HMR to changes in autonomic functions could be low because the HMR was already reduced in more than 90% of patients with early PD. Meanwhile, the WR represents a difference between the eHMR and the dHMR, and we think it can show even more subtle changes between the two parameters. However, there is a need to investigate further whether there is a relationship between changes in the WR and autonomic functions, even in PD patients with a normal HMR.

As there was no consensus of the normal range in the WR, we classified the patients into three groups based on the WR of the subjects in this study to identify the trends of other parameters according to the WR. In spite of the difference of the WR in the three groups, the eHMR and the dHMR showed similar values, and there was no statistically significant difference between the groups. The frequency of OH was significantly increased from group 1 to group 3 (p = 0.011). The dSysPr and the dDiaPr were significantly higher in group 3 (p = 0.016 and p = 0.018), and those parameters had a weak positive correlation with the WR (r = 0.320 and r = 0.275). OH is known to be influenced by age, and it increases in frequency with age. In addition, age and H&Y stage were significantly different between group 1 and group 3 in this study. Because these factors can affect the result of OH related factors, we did ANCOVA with age and H&Y stage as covariates. ANCOVA also revealed that dSysPr and dDiaPr were still significantly larger in group 3 than in another group. In addition, there was no difference in age between group 2 and group 3, but there was a significant difference in the dSysPr and the dDiaPr between them. These facts suggested that the dSysPr and the dDiaPr were different between groups and had an association with the WR regardless of age or H&Y stage. There was a significant correlation between the WR and OH related parameters, but the strength of the association was weak. Although the HUT test to assess OH is a representative and the most common test to evaluate an autonomic function, the status of autonomic function is a very complicated process, and the HUT test mainly represents the baroreflex function [36]. Therefore, we further analyzed the CASS test performed in 20 patients. The CASS test is used to grade the severity of autonomic impairment and fully quantifies the autonomic functions, and the total score has a direct clinical meaning because it classifies the generalized dysautonomia as mild, moderate, or severe [37]. It is difficult to make a definite conclusion because of the small number of patients in this study, but the result was quite interesting. There was a significant difference in the severity of dysautonomia among the three groups, and the frequency of patients with severe symptoms increased in group 3 compared to the other groups (p = 0.014). The total score of the CASS was also the highest in group 3 (p = 0.006). In the CASS test, the cardiosympathetic and total scores were moderately correlated with the WR (r = 0.456 and r = 0.555), and the strength of the relationship was more strong than that of the HUT test. These results suggested that the WR might be associated with dysautonomia and might give information on the status of an autonomic function in patients with PD. However, further study with a larger population is needed. As mentioned earlier, the normal range of the WR has not yet been determined, and various studies have used various normal ranges [12, 22, 26, 38]. To predict OH in PD patients with a decreased HMR, we obtained the optimal cutoff value of the WR by ROC analysis. The cutoff value of 26% was calculated with moderate accuracy (AUC: 0.725, p < 0.001). This cutoff value was similar to previous studies that suggested about 30% as the cutoff value [22, 26, 38].

In this study, the WR of I-123 MIBG cardiac scans were not associated with the dopamine transporter state of the F-18 FP-CIT PET. The SFV and SUR are not significantly different among the three groups (p = 0.455 and p = 0.436), and the two parameters were not correlated with WR (p = 0.360 and p = 0.318). This is consistent with a previous study. Chiaravalloti A, *et al*. reported that there were no statistically significant relationships between the imaging parameters of the I-123 MIBG cardiac scans and the I-123 FP-CIT scans [39]. They suggested that the dysfunction of the dopaminergic and noradrenergic systems were not related in patients with PD. Synucleinopathy, including PD, is known to be a multisystemic disease and can induce both the nigrostriatal neuronal degeneration and autonomic neuronal degeneration independently [8, 9]. These facts supported our results, which showed that there was no association between imaging parameters of I-123 MIBG cardiac and F-18 FP-CIT PET scans.

Recently, it was reported that mutations in several genes, such as the glucocerebrosidase (GBA), Leucine-rich repeat kinase 2 and α-synuclein gene mutations, associated with PD. Especially, non-motor symptoms have been reported to be more severe in PD patients with GBA mutations than in idiopathic PD [40]. There were studies on the association between GBA mutation and I-123 MIBG scan and reported inconsistent results. One study has reported that cardiac uptake in the I-123 MIBG scan was more reduced in the GBA mutation group [41], but the other group has not identified any associations [42]. The present study does not include the association of the gene mutations, further research on the effects of gene mutations is necessary.

The present study has some limitations. First, this is a retrospective analysis with a small number of patients. Therefore, further study is needed with a larger cohort. Second, in general, the HMR is reduced in PD patients, but it is known that some PD patients can show normal values. In this study, we selected the patients with a decreased HMR (less than 1.8) to improve the diagnostic accuracy of PD and to minimize the effect of the HMR due to a mixture of normal and abnormal values. Therefore, a selection bias may exist, but the effect of bias is not expected to be significant because only 10–20% of PD patients can show a normal range of the HMR [8]. Third, there was no advanced PD patients more than stage 3 on the H&Y stage in enrolled subjects of this study. This may be due to the characteristics of a retrospective study conducted mainly in patients who completed initial workup for parkinsonism. Although further evaluation is needed in more advanced PD patients, it is still considered to be significant results in mild to moderate patients with PD. Finally, selection bias may exist in this study because only 20 of the 60 patients performed the CASS test. However, the decision of whether to perform the CASS test was chosen by the patients and not by the researcher.

In conclusion, we evaluated the clinical meaning of the WR on I-123 MIBG cardiac scans in this study, the WR was associated with autonomic dysfunction in patients with PD. I-123 MIBG cardiac scans are thought to be a good method to evaluate not only the differential diagnosis of PD but also the degree of autonomic dysfunction.
